# Quorum sensing protects bacterial co-operation from exploitation by cheats

**DOI:** 10.1038/ismej.2015.232

**Published:** 2016-01-08

**Authors:** Richard C Allen, Luke McNally, Roman Popat, Sam P Brown

**Affiliations:** 1 Centre for Immunity, Infection and Evolution, School of Biological Sciences, University of Edinburgh, Edinburgh, UK

## Abstract

Quorum sensing (QS) is a cell–cell communication system found in many bacterial species, commonly controlling secreted co-operative traits, including extracellular digestive enzymes. We show that the canonical QS regulatory architecture allows bacteria to sense the genotypic composition of high-density populations, and limit co-operative investments to social environments enriched for co-operators. Using high-density populations of the opportunistic pathogen *Pseudomonas aeruginosa* we map per-capita signal and co-operative enzyme investment in the wild type as a function of the frequency of non-responder cheats. We demonstrate mathematically and experimentally that the observed response rule of ‘co-operate when surrounded by co-operators' allows bacteria to match their investment in co-operation to the composition of the group, therefore allowing the maintenance of co-operation at lower levels of population structuring (that is, lower relatedness). Similar behavioural responses have been described in vertebrates under the banner of ‘generalised reciprocity'. Our results suggest that mechanisms of reciprocity are not confined to taxa with advanced cognition, and can be implemented at the cellular level via positive feedback circuits.

## Introduction

The co-operative provision of help to other individuals is a ubiquitous feature of life, from viruses to vertebrates (Turner and Chao, 1999; Bshary and Grutter, 2002; Griffin *et al.*, 2004; Rand *et al.*, 2009; Melis *et al.*, 2011). Across biological scales, co-operative individuals face the challenge of competition with non-co-operative ‘cheats' that reap the rewards of co-operation without paying the full costs (Ghoul *et al.*, 2014). If individuals have fixed strategies (constitutively co-operative or non-co-operative), then co-operators can only outcompete cheats if any net costs of co-operation are sufficiently offset by increased rates of interaction with fellow co-operators (positive genetic assortment or relatedness, Hamilton, 1964; Frank, 1998).

Although much theory has been built on the assumption of constitutive strategies, behavioural plasticity in social traits is increasingly recognised as the norm, not only in vertebrates (Rutte and Taborsky, 2007), but also in microbes (Kuemmerli and Brown, 2010; Parkinson *et al.*, 2011; Xavier *et al.*, 2011) and even in viruses interacting with conspecifics (Leggett *et al.*, 2013). The importance of behavioural control of co-operative effort has been emphasised since Trivers' (1971) pivotal work on reciprocity highlighted that behavioural feedbacks can allow individuals to match their investment in co-operation to the investment of others (that is, creating phenotypic assortment), and thus protect co-operative strategies from exploitation even in well-mixed groups (Trivers, 1971; Nowak and Sigmund, 1998; Pfeiffer *et al.*, 2005; Fletcher and Zwick, 2006). Mechanisms of reciprocity are often suggested to be cognitively complex (Milinski and Wedekind, 1998; Stevens and Hauser, 2004). However, one form of reciprocity, which has been suggested to be simple enough to be achievable by most organisms, is ‘generalised reciprocity' (Pfeiffer *et al.*, 2005). This form of reciprocity works by individuals increasing their level of co-operation as there are more co-operators in their group, removing the requirement for the recognition of individuals or extensive memorisation of past events (Pfeiffer *et al.*, 2005), and has been observed in both humans (Stanca, 2009) and rats (Rutte and Taborsky, 2007).

In bacteria, co-operative interactions are commonly mediated by secreted factors that individual cells produce at a cost to provide shared benefits for a local neighbourhood of cells, for example, iron-scavenging siderophores (Griffin *et al.*, 2004; Kuemmerli and Brown, 2010) or secreted digestive enzymes (Diggle *et al.*, 2007; Sandoz *et al.*, 2007). The cost of secreting proteins is considerable, and has resulted in selection for cheaper amino-acid residues in secreted proteins (Nogueira *et al.*, 2009). It has been suggested that quorum sensing (QS), a common regulatory architecture governing secreted protein production, has evolved in part to restrict secretion investments when they are inefficient (Redfield, 2002; Hense *et al.*, 2007; Darch *et al.*, 2012; Cornforth *et al.*, 2014). We argue that the efficiency gains of QS extend to the context of genetically mixed groups.

QS is a form of cell–cell communication observed across diverse bacterial species (Rutherford and Bassler, 2012). Individuals secrete diffusible signals into the environment and regulate gene expression in response to the concentration of signal. Secreted factors are over-represented in the QS regulon both in *Pseudomonas aeruginosa* (Schuster and Peter Greenberg, 2006; Gilbert *et al.*, 2009; Popat, Cornforth, *et al.*, 2015) and other bacteria (Antunes *et al.*, 2007; Barnard *et al.*, 2007), and therefore non-responsive QS mutants function as social cheats by not producing shared secreted proteins (Diggle *et al.*, 2007; Sandoz *et al.*, 2007).

**Figure 1:**
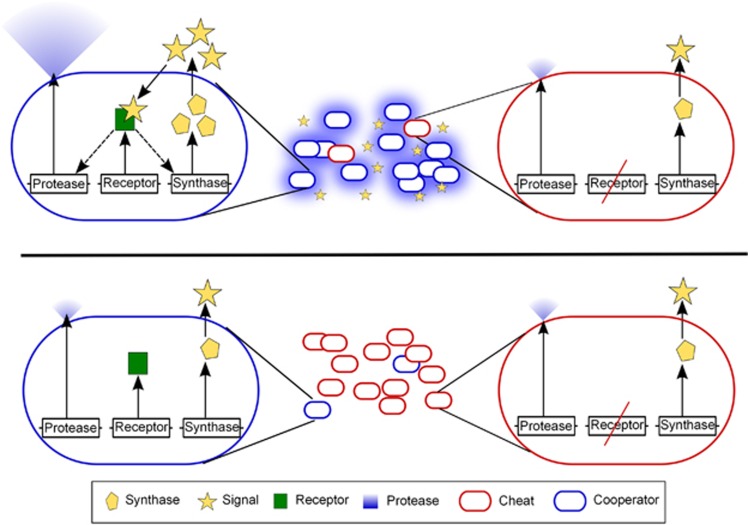
QS network architecture produces the generalised reciprocity rule of ‘co-operate when surrounded by co-operators'. (Top) When co-operators (blue) are common positive signal autoregulation in co-operators leads to high environmental signal concentrations. In response to high signal concentration co-operators express more secreted enzyme, increasing population yield. Cheats (red) lack a signal receptor, they do not respond to signal and so do not express signal or secreted enzyme above basal levels. They are able to exploit co-operators, increasing in frequency. (Bottom) When co-operators are rare, there are few cells undergoing positive feedback in signal production (autoregulation) so signal levels remain low. Co-operators invest little in protease production (acting as phenotypic cheats), keeping costs of co-operation low and reducing exploitation by cheats.

A critical design feature in most QS regulatory networks is positive autoregulation of signal production (Figure 1), a coupling between signal production and signal detection (Goryachev, 2009). Signal responsive wild-type (WT) individuals increase both their rate of signal (Seed *et al.*, 1995) and secreted enzyme (Pearson *et al.*, 1994) production in response to higher environmental signal concentrations, whereas non-responsive mutants (cheats) produce minimal and invariant quantities of signal and enzyme secretions (Figure 1). We suggest that the positive feedback loop governing signal production in the wild type will cause correlations between signal concentrations and the frequency of WT co-operators (genetic composition) in mixed populations, providing a cue to the genotypic composition of the population (Figure 1). The wild-type QS response of increasing enzyme secretions with signal concentration (Pearson *et al.*, 1994) will then lead to co-operative investment, increasing with the frequency of WT co-operators in the population, the outcome of generalised reciprocity. We therefore predict that the native QS architecture will limit the ability of non-responder cheats to socially exploit the wild type in a high-density environment.

A literature review shows that non-responsive cheater mutants (*ΔlasR* mutants) show huge variation in frequency in *P. aeruginosa*, both in samples from infections and experimental evolution studies, varying from 0 to 100% per population (Table 1). As these non-responsive mutants produce little to no signal, this suggests that cheater frequency will be at least as important as population density in determining the signal concentration that a WT co-operator experiences (as at high cheat frequency little signal will be present). The resulting correlation between signal concentration and cheater frequency will allow signal concentration to act as a reliable cue of cheater frequency, thus creating the potential for generalised reciprocity mediated by QS to protect co-operators from exploitation by cheaters.

Building on existing theory (Taylor and Day, 2004; Takezawa and Price, 2010; Cavaliere and Poyatos, 2013) we present mathematical models predicting that generalised reciprocity mediated by QS signals protects public goods investments from exploitation by cheats that are unresponsive to signal. We test this hypothesis by manipulating strain mixing in the opportunistic pathogen and model QS bacterium *P. aeruginosa*. We demonstrate that the wild-type strategy of co-operative investment conditional on autoregulated signal concentration increases the range of conditions (degree of population structure and co-operator frequency) where the regulated co-operative trait can be maintained in the face of non-responsive genetic cheats

## Materials and methods

### Assessing the co-operative phenotype of populations

WT *P. aeruginosa* (PAO1) and an isogenic *lasR* knockout (*ΔlasR*) were used as genetic co-operators and cheats respectively (Diggle *et al.*, 2007). The WT was marked with a *luxCDABE* cassette under the control of the *lasB* promoter (made using the mini CTXLux plasmid, Becher and Schweizer, 2000). The WT and *ΔlasR* strains were mixed at varying proportions and used to seed cultures at an optical density (600 nm) of 0.01. Planktonic cultures were grown in 96-well plates (200 μl volumes) for 6 h in a defined QS medium (Popat *et al.*, 2012) requiring QS-regulated elastase for growth to high density. All *P. aeruginosa* cultures were mixed, by shaking at 250 rpm with an orbit of 37 mm allowing mixing of small volumes (Duetz, 2007) to reduce population structure. After 6 h incubation at 37 °C (cultures produce most signal and response at this time), cultures were centrifuged to separate supernatant and cells. The remaining cell suspension was mixed with equal parts 1:1 Luria-Bertani broth:glycerol and frozen at −80 °C, whereas supernatant was filter sterilised (0.22 μm pore). Frozen populations were later defrosted on ice and frequency was assayed by vortexing, diluting and plating cultures on Luria-Bertani agar plates, and then manually counting colonies using luminescence to distinguish WT and *ΔlasR* colonies. During the counting process the experimenter was blind to treatment.

For measurements of protease activity, 50 μl of sterile supernatant was added to 450 μl of elastin congo red buffer (100 mM Tris, 1 mM CaCl_2_, pH=7) containing 20 mg ml^−1^ elastin congo red (Ohman *et al.*, 1980). Tubes were incubated horizontally with shaking at 150 rpm and 37 °C for 18 h. After incubation, supernatant was removed and the absorbance at 495 nm was read. To determine signal concentrations, filtered culture supernatent was diluted 1/100 in Luria-Bertani broth and mixed in equal concentrations with exponentially growing bioreporter strains at OD 0.1 (600 nm) in Luria-Bertani broth. Synthetic signal at various concentrations was treated similarly to create a calibration curve. Signal bioreporters were grown for 3 h at 37 °C taking reads of optical density and luminescence every 15 min. When luminescence was at a high level (1.75 and 3 h growth for C12 and C4, respectively) a calibration curve was fitted and used to calculate signal concentrations in experimental samples. Bio-reporters were S17-1 *Escherichia coli* containing either the p56536 or pSB1142 plasmids (Winson *et al.*, 1998), which luminesce in response to short and long chain AHLs, respectively.

### Competition experiments

Mixed populations of WT and *ΔlasR* were set up as before, with the following modifications. The WT was marked with the *luxCDABE* cassette in a constitutively active (promoterless) position (Popat *et al.*, 2012). Mixed cultures were grown for 40 h in two 96-well plates (plate did not affect competition). To exclude the effect of drying during extended incubation treatments samples were randomly allocated into the central wells of the plates, outer wells contained growing cultures to minimise effects of oxygen gradients but were not used for analysis. Before and after growth, cells were frozen and later defrosted to ascertain proportions and colony-forming units (CFU) as before. Relative fitness of the WT was calculated as: *X*_1_(1−*X*_0_)/(*X*_0_ (1−*X*_1_)) where *X*_0_ and *X*_1_ are the proportions of WT cells at the start and end of competition, respectively (Ross-Gillespie *et al.*, 2007). The change in proportion of WT over competition can be seen in Supplementary Figure S3.

### Statistics

All statistical analyses were performed in R (R Core Team 2013). Signal and protease phenotype data were modelled as a function of WT proportion at 6 h (*X*_1_):


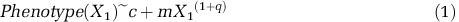


F-tests were used to compare models to assess whether additional terms increased fit, until a minimal model was reached. Reported *P*-values are taken from the minimal model. There was slight variation in the optical density of the samples used to measure phenotypes, but this was always removed from the model due to lack of explanatory power based on F-tests. The statistical model for protease production was used for the function of co-operative effort with WT frequency for analysis in Figure 3.

**Figure 2:**
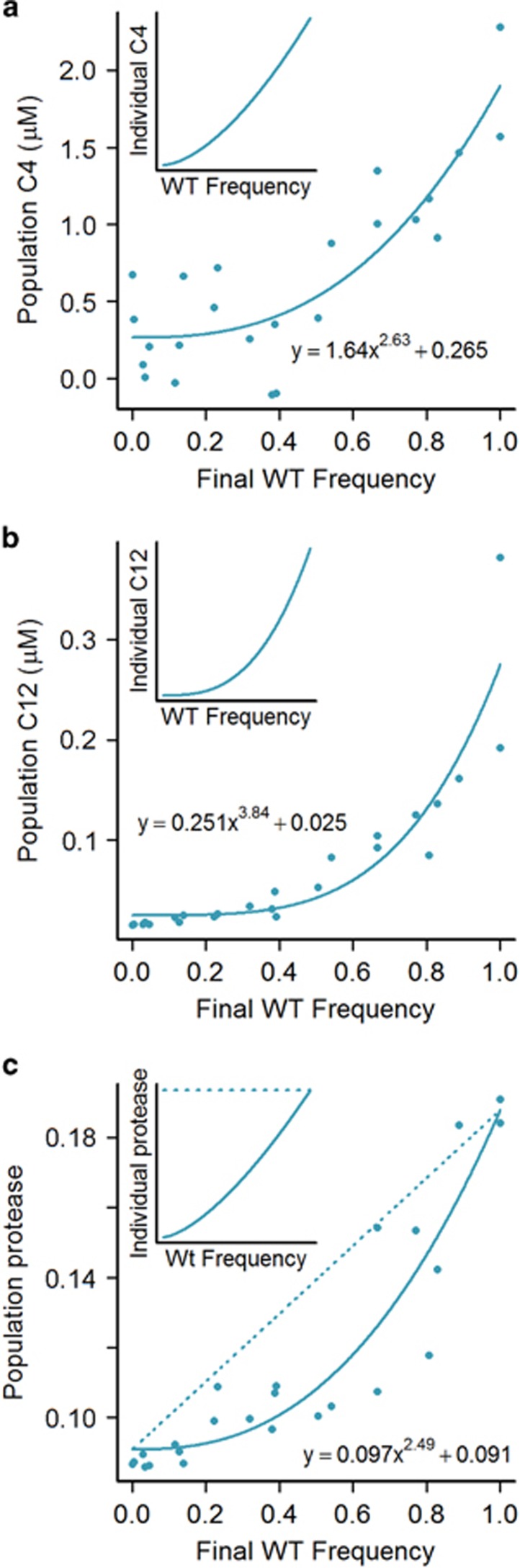
Per-capita signal and secreted enzyme investment increases with wild-type co-operator frequency. Mixed populations of cheats and co-operators were grown for 6 h in quorum-sensing media. After growth, AHL signal and secreted protease concentrations were measured for the population. Plate counts were used to determine co-operator proportion at 6 h. Equations for fitted models are presented on the graphs. Insets show a transformation of the fitted models to show the change in per-capita WT phenotype. (**a**) Total 3-oxo-C12 N- acyl HSL concentration (inset, per WT individual). (**b**) Total C4 N-acyl HSL concentration (inset, per WT individual). (**c**) Total protease (elastase) concentration (inset, per WT individual), the equivalent constitutive response (constant maximal WT investment) is shown as dashed lines.

For competition data, growth rate was calculated based on the CFU of each strain before and after competition as (final CFU–initial CFU)/intital CFU. Linear mixed-effects models were used to model the log of strain growth rates as a function of individual and average group co-operation (taken from the curves in Figure 2c), with competition well included as a random effect. We initially fit a model with effects of individual co-operation, individual co-operation squared, average group co-operation and average group co-operation squared. Model simplification, combined with Wald tests, showed that although the quadratic term for average group co-operation did not significantly improve model fit (effect of group co-operation squared: F_1,52_=2.52, *P*>0.05), all other terms were significant in the reduced model (effect of group co-operation: F_1,53_=134.33, *P*<0.0001; effect of individual co-operation: F_1,49_=210.79, *P*<0.0001; effect of individual co-operation squared: F_1,49_=50.16, *P*<0.0001). The final fitted form of the statistical model was


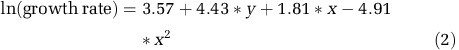


where *x* is individual co-operation (equal to (WT frequency)^1.49^ for the WT and zero for the *ΔlasR* mutant), and *y* is group co-operation (equal to (WT frequency)^2.49^).

### Theoretical framework

We first consider a scenario where co-operators invest a constant amount in co-operation regardless of the composition of their group. For this scenario of constitutive co-operative effort the payoff is


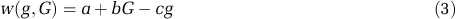


where *g* is an individual's breeding value (1=co-operator, 0=cheat), *G* is the mean breeding value of the population, *a* is the intrinsic payoff, *b* is the benefit of co-operation and *c* is the cost of co-operation. From this we obtain the covariance between fitness and breeding value (that is, the expected change in genotype frequency owing to selection) as


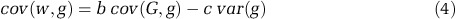


Dividing through by *var(g)*, the variance in breeding value, we get the regression of fitness on breeding value (*β*_*w,g*_), which must be positive for the selection of co-operation


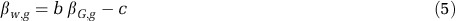


where *β*_*G,g*_ is the regression of average group genotype on individual genotype (that is, relatedness). We will assume that founders are binomially distributed into subpopulations with *n* founders from a total population, which has proportion *p* co-operators. The regression of group genotype on individual genotype can then be simplified as follows:


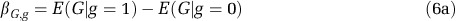



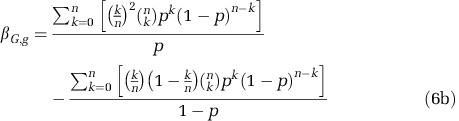



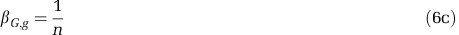


Substituting equation (6c) into equation (5) and rearranging gives us equation (19) from the results. We next consider a scenario where co-operators increase their relative investment linearly from 0 when no co-operators are present to 1 when their group is entirely co-operators. For these assumptions the payoff for a plastic co-operator is


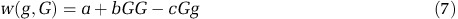


giving the following covariance between fitness and breeding value


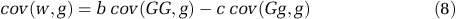


Dividing by *var*(*g*) and rearranging.


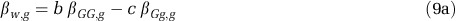



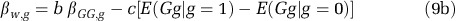



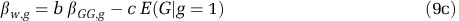


Again assuming a binomial distribution of founders into subpopulations the regression coefficient and expectation can be simplified as


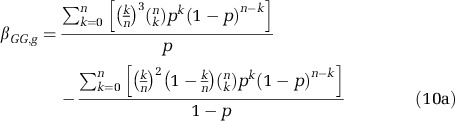



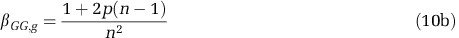


and


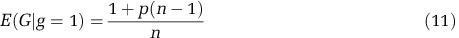


Substituting equations (10b) and (11) into equation (9c) gives equation (21) of the results.

### Metapopulation framework

Metapopulations are commonly used to manipulate structure in microbiology to study social behaviours (Griffin *et al.*, 2004). We used a metapopulation framework to model the effect of signal-meditated generalised reciprocity on the evolution of co-operation. We assume that the metapopulation is structured into an infinite number of groups, each of which is founded by *n* individuals. Given these assumptions, we can calculate the proportion of co-operative individuals that find themselves resident in a group with *k* co-operative founders as


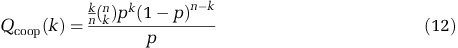


and the proportion of cheats that find themselves resident in a group with *k* co-operative founders as


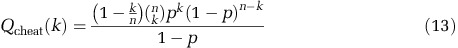


We can then write the difference in fitness between co-operators and cheats as


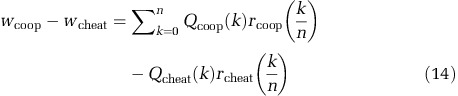


where *r*_*coop*_(*k*/*n*) and *r*_*cheat*_(*k*/*n*) give the growth rates of co-operators and cheats in groups with proportion *k*/*n* co-operators. We compare the behaviour of this fitness difference when *r*_*coop*_(*k*/*n*) and *r*_*cheat*_(*k*/*n*) are calculated first under the natural scenario of QS control of co-operation, and secondly under the assumption of constitutive co-operation. In the scenario of QS-controlled co-operation, the growth rate of co-operators is given as


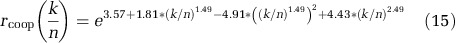


and for cheaters as


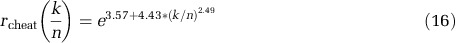


In the scenario of constitutive co-operation, we simply replace all instances of *k*/*n* with 1, giving


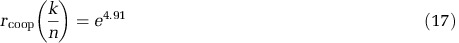


and


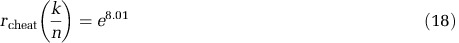


We evaluated the fitness differential for these two scenarios across varying co-operator frequency (*p*) and founder number (*n*) to assess the consequences of signal-mediated generalised reciprocity for the evolution of co-operation.

## Results

### What are the behavioural rules governing signal production and signal response?

The established architecture of QS-controlled secreted enzyme production suggests that increasing the local frequency of wild type (versus signal non-responsive cheats at a fixed population size) will increase the per-capita production rate of both signal (Seed *et al.*, 1995) and secreted protease (Pearson *et al.*, 1994) by WT individuals. To test this prediction and to map the functional forms of the behavioural response, we manipulated the initial frequency of WT co-operators and measured the resulting concentration of the two primary *P. aeruginosa* signal molecules 3-oxo-C12-HSL (*N*-(3-oxododecanoyl)-l-homoserine lactone) and C4-HSL (*N*-(butanoyl)-l-homoserine lactone), plus secreted protease enzyme (a critical *P. aeruginosa* virulence factor and bacterial public good, expressed under positive QS control). Our results are consistent with increases in per-capita C4, C12 and secreted enzyme production by WT individuals when at higher WT frequency. Specifically, in Figure 2 we see that phenotypes at the population level increase as an accelerating function of WT frequency at 6 h, allowing us to reject the null hypothesis of constant per-capita production (exponent of 1). For C4, fitted exponent=2.63, s.e=0.758, *t*_21_=2.148, *P*<0.05. For C12, fitted exponent=3.84, s.e=0.793, *t*_21_=3.576, *P*<0.001. For secreted enzyme, fitted exponent=2.49, s.e=0.492, *t*_21_=3.024, *P*<0.01. Inset plots show the predicted per-capita WT cell phenotype (signal production or co-operative investment). Although QS is often considered to regulate genes in a binary fashion (as a threshold behaviour) our results (Figure 2c and Supplementary Figure S4c) support a view that QS responses are continuous rather than binary, as has also been suggested by a previous study (Darch *et al.*, 2012).

### How does QS control of co-operation shape competition between co-operator and cheat genotypes?

**Figure 3:**
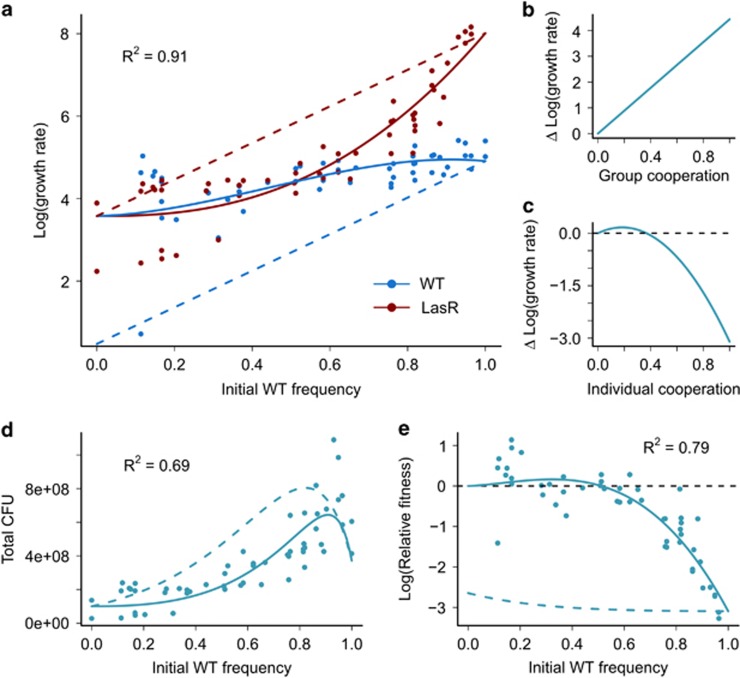
Generalised reciprocity limits social exploitation when WT co-operators are rare. Competition between WT co-operators and cheats (*ΔlasR*) over 40 h in media requiring secreted protease enzyme for growth. (**a**) Log growth rate of the two strains averaged over the length of competition. (**b**) The predicted linear effect of group co-operative effort on log growth rate (effect of group co-operation: *β*=4.43, s.e.=0.26, F_1,53_=134.33, *P*<0.0001). (**c**) The predicted quadratic effect of individual co-operative effort on log growth rate (effect of individual co-operation: *β*=1.81, s.e.=0.54, F_1,49_=210.79, *P*<0.0001; effect of individual co-operation squared: *β*=–4.91, s.e.=0.69, F_1,49_=50.16, *P*<0.0001). (**d**) How population yield (CFU/100 μl) varies with WT frequency. (**e**) How WT relative fitness varies with WT frequency. All solid lines are fits of our model to the data shown in (**a**) assuming co-operative effort varies with WT frequency according to the solid fits in Figure 2c. Dashed lines are predictions for constitutive co-operative effort (assuming co-operative effort varies with WT frequency according to dashed lines in (2c), individual points are direct measures of growth rate, yield and relative fitness measured from competition experiments.

To ask whether the native functional response ‘co-operate when surrounded by co-operators' (Figure 2c) protects protease enzyme secretion from exploitation, we performed 40 h competition experiments between WT co-operators and non-responder cheats at varying initial frequencies. The growth rate of both strains increased with the initial WT frequency (Figure 3a). To relate growth rate to the underlying costs and benefits of co-operative enzyme secretion, we use our estimates of individual and collective co-operative effort as a function of WT frequency at 6 h (Figure 2c). We fit a linear mixed-effect model of the log of growth rate (Figure 3a) as a function of individual (Figure 2c, inset) and collective (Figure 2c, main) enzyme secretion phenotypes. The analysis partitions out the effects of mean group phenotype (Figure 3b) and individual phenotype (Figure 3c). Although the growth rate of both strains increases with initial WT frequency (Figure 3a) due to the benefits of group co-operation (Figure 3b), the cheat strain benefits more at high initial WT frequency because secreted enzyme production is individually costly (Figures 3a and c). Fixing co-operative effort as constant (using the dashed line in Figure 2c) allows us to model the growth rate of the two strains given constitutive WT co-operation (dashed lines in Figure 3a).

We can use our fitted model of growth rate to summarise the yield data (measured as CFU) and relative fitness data for the competition experiments in Figures 3d and e (data points and solid lines). These show good agreement with the data capturing the superlinear increase in yield (but showing a reduction in yield at very high initial WT frequencies, see MacLean *et al.*, 2010) and increasing exploitation of the WT when common. When co-operative effort is constitutive (dashed lines), our predictions indicate that yield increases more linearly and the WT is always exploited. Experimentally fixing per-capita WT co-operative effort at a constant (but increased) level through the addition of excess signals gives qualitatively similar results (Supplementary Figure S1).

### What are the implications of signal autoregulation for selection across a metapopulation?

To explore the consequences of QS regulatory control of co-operation, we next build and analyse a simple model of bacterial fitness as a function of their social neighbourhood (Chuang *et al.*, 2009; de Vargas Roditi *et al.*, 2013), with and without QS regulatory control of co-operation, assuming linear costs and benefits of co-operation. Under the assumption of constitutive co-operation, we find that a co-operative strain is under positive selection whenever *b*β_*G*,*g*>*c*_, where *b* and *c* are the benefits of co-operation and *β*_*G*,*g*_ is the coefficient of relatedness (in regression coefficient form), a measure of genetic assortment between co-operators (with breeding value *g*) and their social group (with breeding value *G*). When we assume that WT and cheat strains are allocated at random (binomially) to subpopulations, the relatedness term reduces to the inverse of the number of patch founders, *n* (methods), meaning co-operation will be favoured whenever


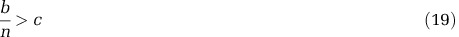


To introduce plastic co-operative effort, we make the conservative assumption that individual co-operative investment increases linearly with the proportion of co-operators (in practice, investment increases even faster with increasing frequency of co-operators, Figure 2c). This assumption implies that the costs of co-operation for an individual increase linearly with the local frequency of co-operators, whereas the per-capita benefits of co-operation increase quadratically (more co-operators and more effort). As a result, the condition for co-operation to be favoured is now


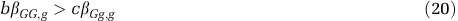


Using the binomial distribution to derive the regression coefficients (methods), and substituting the values for *β*_*Gg*,*g*_ and *β*_*GG,g*_ into equation (20) we see that the condition for QS-controlled co-operation to be favoured is:


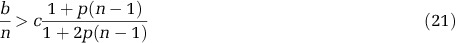


where *p* is the proportion of individuals in the metapopulation that are co-operators (*g*=1). Comparing the right hand sides of inequalities 19 and 21, we can see that co-operation is more easily favoured by selection under signal-mediated generalised reciprocity whenever *P*>0 and *n*>1; that is, so long as some co-operators are present and groups are not clonal. Where *P*=0 and/or *n*=1 the inequalities are identical. Thus, signal-mediated generalised reciprocity is expected to protect co-operative individuals from exploitation by cheaters.

**Figure 4:**
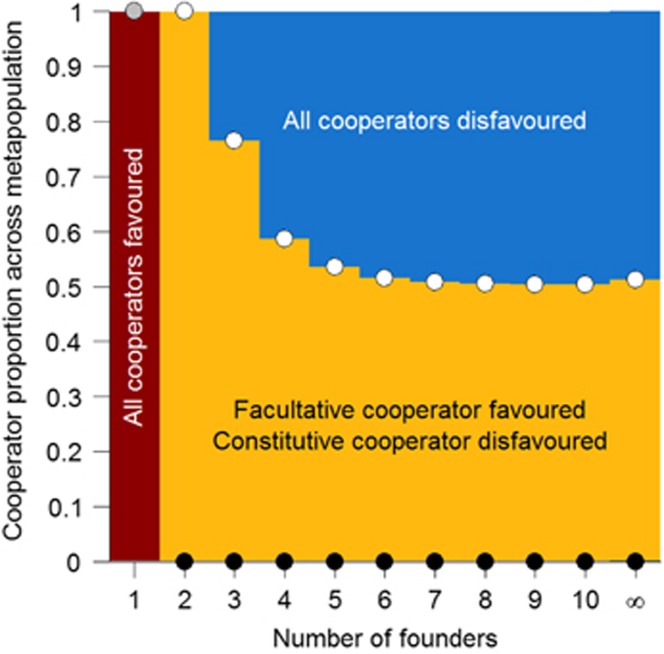
Generalised reciprocity stabilises co-operation over a larger range of conditions in a metapopulation. Selection determined using statistical model fits shown in Figure 3 and the metapopulation framework described in the methods. Dots represent equilibria for constitutive (black), QS-regulated (white), and either form of co-operation (grey). Constitutive co-operation is only favoured when the population has a clonal structure (1 founder per subpopulation). QS-regulated (facultative) co-operation is favoured for lower levels of relatedness, including a well-mixed population (infinite number of founders).

Although our theoretical model suggests that signal-mediated generalised reciprocity helps protect co-operators from exploitation by cheaters in a metapopulation, it makes simplifying assumptions regarding the relationships between strain fitness, and individual and group co-operation. To assess our theoretical predictions, we used our fitted fitness functions (Figure 3) to determine the consequences of signal-mediated generalised reciprocity for the evolution of co-operation in a metapopulation. Our results confirm that generalised reciprocity reduces the level of relatedness necessary for co-operation to be favoured, allowing co-operation to be more readily maintained in non-clonal groups (Figure 4). In Figure 4 the final column shows a metapopulation where subpopulations have an infinite number of founders, this is equivalent to a well-mixed population and thus is identical to the data in Figure 3.

## Discussion

QS bacteria commonly place the control of both secreted signals and proteins under the positive influence of extracellular signal molecule concentration (Antunes *et al.*, 2007; Gilbert *et al.*, 2009; Rutherford and Bassler, 2012). We illustrate that this well-studied mechanism allows bacteria to assess the extent of co-operation in their surrounding population (Figure 2), and respond with a rule similar to generalised reciprocity that can limit exploitation by signal non-responsive cheats (Figures 3 and 4). In comparison with constitutive co-operation, the rule of ‘co-operate when surrounded by co-operators' moderates individual co-operative effort to be closer to the surrounding population phenotype (that is, increasing phenotypic assortment, Fletcher and Zwick, 2006) and reducing exploitation by cheats. Our results in Figure 4 predict that facultative co-operation may lead to co-existence of co-operators and cheats in natural settings.

As can be seen in Figure 2c and Supplementary Figure S4c, when the WT is rare co-operative investment is low. Our analysis in Figure 3c shows that although co-operation at high levels is costly to the individual, a low level of co-operation benefits the individual. We experimentally measure the costs of expression in Supplementary Figures S4b and S4d showing that the low level of QS induction when the WT is rare results in low or negligible costs. This experiment removes the benefits of protease production (there is no protein in the environment), but given the low cost of QS induction at a low level we would see a WT relative fitness >1 if there is any individual benefit of low level QS induction. Individual benefits may be due to preferential access to the low level of protease in the environment, for example, owing to association of protease with the membrane of the producing cell or residual spatial structure as *P. aeruginosa* can form planktonic aggregates (Schleheck *et al.*, 2009). QS controls many genes so other traits such as stress responses may be responsible for individual benefits (García-Contreras *et al.*, 2014; Davenport *et al.*, 2015). Private benefits are not required for our metapopulation results, if we alter our metapopulation framework so that cheats produce the level of protease that is most individually beneficial (corresponding to the maxima of the curve in Figure 3c), we still see a benefit of QS control of public goods (Supplementary Figure S2). In addition, we see an area of positive frequency dependence, producing bistability (Supplementary Figure S2).

The key to generalised reciprocity in the *P. aeruginosa* system (and others sharing the key signal autoregulation design) is the pleiotropic action of the LasR signal receptor protein (Foster *et al.*, 2004; Dandekar *et al.*, 2012). As a transcription factor it determines both the level of signal production and the level of QS-regulated-secreted factors. The *lasR* gene (and thus signal production) resembles a greenbeard mechanism, generating a detectable signal that is mechanistically linked to investment in co-operation (Dawkins, 1989; Eldar, 2011; Biernaskie *et al.*, 2013). We do not demonstrate that the observed response rules are evolutionary stable strategies (Takezawa and Price, 2010; Cavaliere and Poyatos, 2013) and it is possible that signal production could become unlinked from signal response, thus breaking the pleiotropy and the ability for generalised reciprocity to protect the WT from exploitation. In particular, the WT regulatory response appears vulnerable to a coercive-cheating strategy of high signal, low response (Brown and Johnstone, 2001; Eldar, 2011). However, clinical studies commonly isolate low signal, low-response *lasR* mutants (Table 1), and recent experimental evolution suggests that a coercive-signalling phenotype is not readily available to selection (Popat, Pollitt, *et al.*, 2015).

A role is proposed for WT QS to sense the genetic composition of a high-density population; however, it is unknown to what extent this is adaptive in natural populations of *P. aeruginosa*. The functional role of QS is the subject of significant debate, with QS implicated as a device to sense variation in density (Darch *et al.*, 2012), mass transfer (Redfield, 2002; Boedicker *et al.*, 2009) or the spatial patterning of a population (Hense *et al.*, 2007). These environmental factors, along with others, will affect the concentration of signals. Like other hypotheses for functions of QS the importance of QS in sensing genotypic composition will depend on how much variation there is in *ΔlasR* frequency (or similar signal blind mutations) in the environment and clinical setting compared with variation in other factors. Our review of current literature suggests that there are high levels of variation in the proportion of lasR-defective strains in a natural setting (Table 1), with cheat frequencies varying from 0 to 100% within local subpopulations. As large groups with high cheat frequencies will produce very little signal, this suggests that cheat frequency may be at least as important as population density in determining signal concentrations in nature.

The addition of genotype sensing to the list of potential QS functions should not be seen as incompatible with the hypotheses mentioned above. Recent work has argued that using multiple signals allows *P. aeruginosa* to improve resolution of both population density and mass transfer properties (Cornforth *et al.*, 2014). It is possible that wild-type populations growing clonally can use QS to draw sophisticated inferences about the state of their social and physical environment (Diggle *et al.*, 2007; Hense *et al.*, 2007; Darch *et al.*, 2012), and in addition can use the same QS apparatus to reduce social exploitation when confronted with non-responder cheat genotypes (Figures 2–4).

An understanding of QS-regulated secretions takes on an additional importance given the current focus on QS as a therapeutic target (Rasko and Sperandio, 2010; Rutherford and Bassler, 2012; Schuster *et al.*, 2013; Allen *et al.*, 2014). Under the action of QS receptor blocking therapeutics, sensitive strains will behave like non-responder cheats, whereas resistant mutants can potentially maintain wild-type behaviour (Mellbye and Schuster, 2011; Allen *et al.*, 2014), and risk being counter-selected in well-mixed populations, owing to their investment in co-operation. However, our results highlight that resistant co-operators will invest little in co-operation when they arise at a low frequency, expressing a similar phenotype to susceptible strains, leading to relaxed selection for or against resistance (Gerdt and Blackwell, 2014). Similarly, if therapeutics targeting signal supply are used they will reduce the concentration of active signal (Dong *et al.*, 2001; Gutierrez *et al.*, 2009). The perceived proportion of co-operators will then be lower, so genetic co-operators will co-operate less, again relaxing selection for or against genetic co-operators, potentially leading to the maintenance of elevated virulence (Köhler *et al.*, 2010). Our data highlight the need to understand drug targets in detail to predict the evolutionary consequences of treatment with new therapeutics (Allen *et al.*, 2014; Ross-Gillespie *et al.*, 2014; Vale *et al.*, 2014).

Plasticity is commonly observed in bacterial species, and the importance of plasticity in co-operative phenotypes is increasingly becoming apparent (Kuemmerli *et al.*, 2009; de Vargas Roditi *et al.*, 2013). What then can behavioural ecology tell us about plasticity in microbial co-operation (Inglis *et al.*, 2014)? Our results show that a behavioural rule of ‘co-operate if surrounded by co-operators' is found in bacteria. This approximates generalised reciprocity: co-operate based on previous co-operative experiences regardless of identity of partners (Pfeiffer *et al.*, 2005). Generalised reciprocity has been reported in humans (Stanca, 2009) and rats (Rutte and Taborsky, 2007), and the small cognitive demands of generalised reciprocity suggest that it will be more universally applicable. We find that bacteria implement a similar social rule, suggesting that generalised reciprocity is not confined to taxa with advanced cognition, and can be implemented at the cellular level via simple positive feedback regulatory circuits.

## Figures and Tables

**Table 1:** *lasR*-defective mutants show large variation in local frequency

*ΔlasR proportion*	*Environment*	*Reference*
40–85%	Cystic fibrosis patients	(Köhler *et al.*, 2010)
0–100%	Cystic fibrosis Patients	(Hoffman *et al.*, 2009)
2–35%	Mouse models of infection	(Rumbaugh *et al.*, 2009)
0–40%	Experimental evolution *in vitro*	(Sandoz *et al.*, 2007)
0–80%	Experimental evolution *in vitro*	(Dandekar *et al.*, 2012)

## References

[bib1] Allen RC, Popat R, Diggle SP, Brown SP. (2014). Targeting virulence: can we make evolution-proof drugs? Nat Rev Microbiol 12: 300–308. 24625893 10.1038/nrmicro3232

[bib2] Antunes LCM, Schaefer AL, Ferreira RBR, Qin N, Stevens AM, Ruby EG et al. (2007). Transcriptome analysis of the *Vibrio fischeri* LuxR-LuxI regulon. J Bacteriol 189: 8387–8391. 17827287 10.1128/JB.00736-07 PMC2168698

[bib3] Barnard AML, Bowden SD, Burr T, Coulthurst SJ, Monson RE, Salmond GPC. (2007). Quorum sensing, virulence and secondary metabolite production in plant soft-rotting bacteria. Philos Trans R Soc Lond B Biol Sci 362: 1165–1183. 17360277 10.1098/rstb.2007.2042 PMC2435580

[bib4] Becher A, Schweizer HP. (2000). Integration-proficient *Pseudomonas aeruginosa* vectors for isolation of single-copy chromosomal lacZ and lux gene fusions. Biotechniques 29: 948–950, 952. 11084852 10.2144/00295bm04

[bib5] Biernaskie JM, Gardner A, West SA. (2013). Multicoloured greenbeards, bacteriocin diversity and the rock-paper-scissors game. J Evol Biol 26: 2081–2094. 23980628 10.1111/jeb.12222

[bib6] Boedicker JQ, Vincent ME, Ismagilov RF. (2009). Microfluidic confinement of single cells of bacteria in small volumes initiates high-density behavior of quorum sensing and growth and reveals its variability. Angew Chem Int Ed Engl 48: 5908–5911. 19565587 10.1002/anie.200901550 PMC2748941

[bib7] Brown SP, Johnstone RA. (2001). Cooperation in the dark: signalling and collective action in quorum-sensing bacteria. Proc Biol Sci 268: 961–965. 11370970 10.1098/rspb.2001.1609 PMC1088694

[bib8] Bshary R, Grutter AS. (2002). Asymmetric cheating opportunities and partner control in a cleaner fish mutualism. Anim Behav 63: 547–555.

[bib9] Cavaliere M, Poyatos JF. (2013). Plasticity facilitates sustainable growth in the commons. J R Soc Interface 10: 20121006. 23365195 10.1098/rsif.2012.1006 PMC3627111

[bib10] Chuang JS, Rivoire O, Leibler S. (2009). Simpson's paradox in a synthetic microbial system. Science 323: 272–275. 19131632 10.1126/science.1166739

[bib11] Cornforth DM, Popat R, McNally L, Gurney J, Scott-Phillips TC, Ivens A et al. (2014). Combinatorial quorum sensing allows bacteria to resolve their social and physical environment. Proc Natl Acad Sci USA 111: 4280–4284. 24594597 10.1073/pnas.1319175111 PMC3964068

[bib12] Dandekar AA, Chugani S, Greenberg EP. (2012). Bacterial quorum sensing and metabolic incentives to cooperate. Science 338: 264–266. 23066081 10.1126/science.1227289 PMC3587168

[bib13] Darch SE, West SA, Winzer K, Diggle SP. (2012). Density-dependent fitness benefits in quorum-sensing bacterial populations. Proc Natl Acad Sci USA 109: 8259–8263. 22566647 10.1073/pnas.1118131109 PMC3361460

[bib14] Davenport PW, Griffin JL, Welch M. (2015). Quorum sensing is accompanied by global metabolic changes in the opportunistic human pathogen pseudomonas aeruginosa. J Bacteriol 197: 2072–2082. 25868647 10.1128/JB.02557-14 PMC4438216

[bib15] Dawkins R. (1989) The Selfish Gene, 2nd edn. Oxford Paperbacks: Oxford, New York.

[bib16] de Vargas Roditi L, Boyle KE, Xavier JB. (2013). Multilevel selection analysis of a microbial social trait. Mol Syst Biol 9: Article 684. 10.1038/msb.2013.42 PMC3779802 23959025

[bib17] Diggle SP, Griffin AS, Campbell GS, West SA. (2007). Cooperation and conflict in quorum-sensing bacterial populations. Nature 450: 411–414. 18004383 10.1038/nature06279

[bib18] Dong YH, Wang LH, Xu JL, Zhang HB, Zhang XF, Zhang LH. (2001). Quenching quorum-sensing-dependent bacterial infection by an N-acyl homoserine lactonase. Nature 411: 813–817. 11459062 10.1038/35081101

[bib19] Duetz WA. (2007). Microtiter plates as mini-bioreactors: miniaturization of fermentation methods. Trends Microbiol 15: 469–475. 17920276 10.1016/j.tim.2007.09.004

[bib20] Eldar A. (2011). Social conflict drives the evolutionary divergence of quorum sensing. Proc Natl Acad Sci USA 108: 13635–13640. 21807995 10.1073/pnas.1102923108 PMC3158151

[bib21] Fletcher JA, Zwick M. (2006). Unifying the theories of inclusive fitness and reciprocal altruism. Am Nat 168: 252–262. 16874634 10.1086/506529

[bib22] Foster KR, Shaulsky G, Strassmann JE, Queller DC, Thompson CRL. (2004). Pleiotropy as a mechanism to stabilize cooperation. Nature 431: 693–696. 15470429 10.1038/nature02894

[bib23] Frank SA. (1998) Foundations of social evolution. Princeton University Press: Princeton, NJ, USA.

[bib24] García-Contreras R, Nuñez-López L, Jasso-Chávez R, Kwan BW, Belmont JA, Rangel-Vega A et al. (2014). Quorum sensing enhancement of the stress response promotes resistance to quorum quenching and prevents social cheating. ISME J 9: 115–125. 24936763 10.1038/ismej.2014.98 PMC4274435

[bib25] Gerdt JP, Blackwell HE. (2014). Competition studies confirm two major barriers that can preclude the spread of resistance to quorum-sensing inhibitors in bacteria. ACS Chem Biol 9: 2291–2299. 25105594 10.1021/cb5004288 PMC4201345

[bib26] Ghoul M, Griffin AS, West SA. (2014). Toward an evolutionary definition of cheating. Evolution 68: 318–331. 24131102 10.1111/evo.12266

[bib27] Gilbert KB, Kim TH, Gupta R, Greenberg EP, Schuster M. (2009). Global position analysis of the *Pseudomonas aeruginosa* quorum-sensing transcription factor LasR. Mol Microbiol 73: 1072–1085. 19682264 10.1111/j.1365-2958.2009.06832.x PMC2759405

[bib28] Goryachev AB. (2009). Design principles of the bacterial quorum sensing gene networks. Wiley Interdiscip Rev Syst Biol Med 1: 45–60. 20835981 10.1002/wsbm.27

[bib29] Griffin AS, West SA, Buckling A. (2004). Cooperation and competition in pathogenic bacteria. Nature 430: 1024–1027. 15329720 10.1038/nature02744

[bib30] Gutierrez JA, Crowder T, Rinaldo-Matthis A, Ho M-C, Almo SC, Schramm VL. (2009). Transition state analogs of 5′-methylthioadenosine nucleosidase disrupt quorum sensing. Nat Chem Biol 5: 251–257. 19270684 10.1038/nchembio.153 PMC2743263

[bib31] Hamilton W. (1964). Genetical evolution of social behaviour I. J Theor Biol 7: 1–16. 5875341 10.1016/0022-5193(64)90038-4

[bib32] Hense BA, Kuttler C, Muller J, Rothballer M, Hartmann A, Kreft J-U. (2007). Does efficiency sensing unify diffusion and quorum sensing? Nat Rev Microbiol 5: 230–239. 17304251 10.1038/nrmicro1600

[bib33] Hoffman LR, Kulasekara HD, Emerson J, Houston LS, Burns JL, Ramsey BW et al. (2009). *Pseudomonas aeruginosa* lasR mutants are associated with cystic fibrosis lung disease progression. J Cyst Fibros Off J Eur Cyst Fibros Soc 8: 66–70. 10.1016/j.jcf.2008.09.006 PMC2631641 18974024

[bib34] Inglis RF, West S, Buckling A. (2014). An experimental study of strong reciprocity in bacteria. Biol Lett 10: 20131069. 24501270 10.1098/rsbl.2013.1069 PMC3949375

[bib35] Köhler T, Perron GG, Buckling A, van Delden C. (2010). Quorum sensing inhibition selects for virulence and cooperation in *Pseudomonas aeruginosa*. PLoS Pathog 6: e1000883. 20463812 10.1371/journal.ppat.1000883 PMC2865528

[bib36] Kuemmerli R, Brown SP. (2010). Molecular and regulatory properties of a public good shape the evolution of cooperation. Proc Natl Acad Sci USA 107: 18921–18926. 20944065 10.1073/pnas.1011154107 PMC2973908

[bib37] Kuemmerli R, Jiricny N, Clarke LS, West SA, Griffin AS. (2009). Phenotypic plasticity of a cooperative behaviour in bacteria. J Evol Biol 22: 589–598. 19170825 10.1111/j.1420-9101.2008.01666.x

[bib38] Leggett HC, Benmayor R, Hodgson DJ, Buckling A. (2013). Experimental evolution of adaptive phenotypic plasticity in a parasite. Curr Biol 23: 139–142. 23246405 10.1016/j.cub.2012.11.045

[bib39] MacLean RC, Fuentes-Hernandez A, Greig D, Hurst LD, Gudelj I. (2010). Amixture of ‘cheats' and ‘co-operators' can enable maximal group benefit. PLoS Biol 8: e1000486. 20856906 10.1371/journal.pbio.1000486 PMC2939026

[bib40] Melis AP, Warneken F, Jensen K, Schneider A-C, Call J, Tomasello M. (2011). Chimpanzees help conspecifics obtain food and non-food items. Proc R Soc B-Biol Sci 278: 1405–1413. 10.1098/rspb.2010.1735 PMC3061135 20980301

[bib41] Mellbye B, Schuster M. (2011). The sociomicrobiology of antivirulence drug resistance: a proof of concept. mBio 2: e00131–11. 21990612 10.1128/mBio.00131-11 PMC3190357

[bib42] Milinski M, Wedekind C. (1998). Working memory constrains human cooperation in the Prisoner's Dilemma. Proc Natl Acad Sci USA 95: 13755–13758. 9811873 10.1073/pnas.95.23.13755 PMC24892

[bib43] Nogueira T, Rankin DJ, Touchon M, Taddei F, Brown SP, Rocha EPC. (2009). Horizontal gene transfer of the secretome drives the evolution of bacterial cooperation and virulence. Curr Biol 19: 1683–1691. 19800234 10.1016/j.cub.2009.08.056 PMC2773837

[bib44] Nowak MA, Sigmund K. (1998). Evolution of indirect reciprocity by image scoring. Nature 393: 573–577. 9634232 10.1038/31225

[bib45] Ohman DE, Cryz SJ, Iglewski BH. (1980). Isolation and characterization of *Pseudomonas aeruginosa* PAO mutant that produces altered elastase. J Bacteriol 142: 836–842. 6769912 10.1128/jb.142.3.836-842.1980 PMC294108

[bib46] Parkinson K, Buttery NJ, Wolf JB, Thompson CRL. (2011). A simple mechanism for complex social behavior. PLoS Biol 9: e1001039. 21468302 10.1371/journal.pbio.1001039 PMC3066132

[bib47] Pearson J, Gray K, Passador L, Tucker K, Eberhard A, Iglewski B et al. (1994). Structure of theautoinducer required for expression of pseudomonas-aeruginosa virulence genes. Proc Natl Acad Sci USA 91: 197–201. 8278364 10.1073/pnas.91.1.197 PMC42913

[bib48] Pfeiffer T, Rutte C, Killingback T, Taborsky M, Bonhoeffer S. (2005). Evolution of cooperation by generalized reciprocity. Proc R Soc B Biol Sci 272: 1115–1120. 10.1098/rspb.2004.2988 PMC1559812 16024372

[bib49] Popat R, Cornforth DM, McNally L, Brown SP. (2015). Collective sensing and collective responses in quorum-sensing bacteria. J R Soc Interface 12: 20140882. 25505130 10.1098/rsif.2014.0882 PMC4305403

[bib50] Popat R, Crusz SA, Messina M, Williams P, West SA, Diggle SP. (2012). Quorum-sensing and cheating in bacterial biofilms. Proc R Soc B Biol Sci 279: 4765–4771. 10.1098/rspb.2012.1976 PMC3497100 23034707

[bib51] Popat R, Pollitt EJG, Harrison F, Naghra H, Hong K-W, Chan K-G et al. (2015). Conflict of interest and signal interference lead to the breakdown of honest signaling. Evolution 69: 2371–2383. 26282874 10.1111/evo.12751 PMC4862024

[bib52] Rand DG, Dreber A, Ellingsen T, Fudenberg D, Nowak MA. (2009). Positive interactions promote public cooperation. Science 325: 1272–1275. 19729661 10.1126/science.1177418 PMC2875121

[bib53] Rasko DA, Sperandio V. (2010). Anti-virulence strategies to combat bacteria-mediated disease. Nat Rev Drug Discov 9: 117–128. 20081869 10.1038/nrd3013

[bib54] R Core Team. (2013). R: A Language and Environment for Statistical Computing. R Foundation for Statistical Computing: Vienna, Austria. http://www.R-project.org/.

[bib55] Redfield R. (2002). Is quorum sensing a side effect of diffusion sensing? Trends Microbiol 10: 365–370. 12160634 10.1016/s0966-842x(02)02400-9

[bib56] Ross-Gillespie A, Gardner A, West SA, Griffin AS. (2007). Frequency dependence and cooperation: theory and a test with bacteria. Am Nat 170: 331–342. 17879185 10.1086/519860

[bib57] Ross-Gillespie A, Weigert M, Brown SP, Kümmerli R. (2014). Gallium-mediated siderophore quenching as an evolutionarily robust antibacterial treatment. Evol Med Public Health 2014: 18–29. 24480613 10.1093/emph/eou003 PMC3935367

[bib58] Rumbaugh KP, Diggle SP, Watters CM, Ross-Gillespie A, Griffin AS, West SA. (2009). Quorum sensing and the social evolution of bacterial virulence. Curr Biol 19: 341–345. 19230668 10.1016/j.cub.2009.01.050

[bib59] Rutherford ST, Bassler BL. (2012). Bacterial quorum sensing: its role in virulence and possibilities for its control. Cold Spring Harb Perspect Med 2: a012427. 23125205 10.1101/cshperspect.a012427 PMC3543102

[bib60] Rutte C, Taborsky M. (2007). Generalized reciprocity in rats. PLoS Biol 5: e196. 17608566 10.1371/journal.pbio.0050196 PMC1914408

[bib61] Sandoz KM, Mitzimberg SM, Schuster M. (2007). Social cheating in *Pseudomonas aeruginosa* quorum sensing. Proc Natl Acad Sci USA 104: 15876–15881. 17898171 10.1073/pnas.0705653104 PMC2000394

[bib62] Schleheck D, Barraud N, Klebensberger J, Webb JS, McDougald D, Rice SA et al. (2009). *Pseudomonas aeruginosa* PAO1 preferentially grows as aggregates in liquid batch cultures and disperses upon starvation. Plos One 4: e5513. 19436737 10.1371/journal.pone.0005513 PMC2677461

[bib63] Schuster M, Joseph Sexton D, Diggle SP, Peter Greenberg E. (2013). Acyl-homoserine lactone quorum sensing: from evolution to application. Annu Rev Microbiol 67: 43–63. 23682605 10.1146/annurev-micro-092412-155635

[bib64] Schuster M, Peter Greenberg E. (2006). A network of networks: quorum-sensing gene regulation in *Pseudomonas aeruginosa*. Int J Med Microbiol 296: 73–81. 16476569 10.1016/j.ijmm.2006.01.036

[bib65] Seed P, Passador L, Iglewski B. (1995). Activation of the pseudomonas-aeruginosa lasi gene by lasR and the pseudomonas autoinducer PAI - an autoinduction regulatory hierarchy. J Bacteriol 177: 654–659. 7836299 10.1128/jb.177.3.654-659.1995 PMC176640

[bib66] Stanca L. (2009). Measuring indirect reciprocity: whose back do we scratch? J Econ Psychol 30: 190–202.

[bib67] Stevens JR, Hauser MD. (2004). Why be nice? Psychological constraints on the evolution of cooperation. Trends Cogn Sci 8: 60–65. 15588809 10.1016/j.tics.2003.12.003

[bib68] Takezawa M, Price ME. (2010). Revisiting ‘The Evolution of Reciprocity in Sizable Groups': Continuous reciprocity in the repeated n-person prisoner's dilemma. J Theor Biol 264: 188–196. 20144622 10.1016/j.jtbi.2010.01.028

[bib69] Taylor PD, Day T. (2004). Stability in negotiation games and the emergence of cooperation. Proc R Soc B Biol Sci 271: 669–674. 10.1098/rspb.2003.2636 PMC1691654 15209098

[bib70] Trivers RL. (1971). The evolution of reciprocal altruism. Q Rev Biol 46: 35–57.

[bib71] Turner PE, Chao L. (1999). Prisoner's dilemma in an RNA virus. Nature 398: 441–443. 10201376 10.1038/18913

[bib72] Vale PF, Fenton A, Brown SP. (2014). Limiting damage during Infection: lessons from infection tolerance for novel therapeutics. PLoS Biol 12: e1001769. 24465177 10.1371/journal.pbio.1001769 PMC3897360

[bib73] Winson MK, Swift S, Fish L, Throup JP, Jørgensen F, Chhabra SR et al. (1998). Construction and analysis of luxCDABE-based plasmid sensors for investigating N-acyl homoserine lactone-mediated quorum sensing. FEMS Microbiol Lett 163: 185–192. 9673021 10.1111/j.1574-6968.1998.tb13044.x

[bib74] Xavier JB, Kim W, Foster KR. (2011). A molecular mechanism that stabilizes cooperative secretions in *Pseudomonas aeruginosa*. Mol Microbiol 79: 166–179. 21166901 10.1111/j.1365-2958.2010.07436.x PMC3038674

