# Supplementary material for: Quorum sensing protects bacterial co-operation from exploitation by cheats
Source: ISME J. 2016 Jan 8;10(7):1706–16. doi: 10.1038/ismej.2015.232 (PMC4918439; doi:10.1038/ismej.2015.232)
Supplement: Supplementary Information [file ismej2015232x1.docx]

**Quorum sensing protects bacterial cooperation from exploitation by cheats.**

Richard C. Allen^†^, Luke McNally^†^, Roman Popat, Sam P. Brown*

**
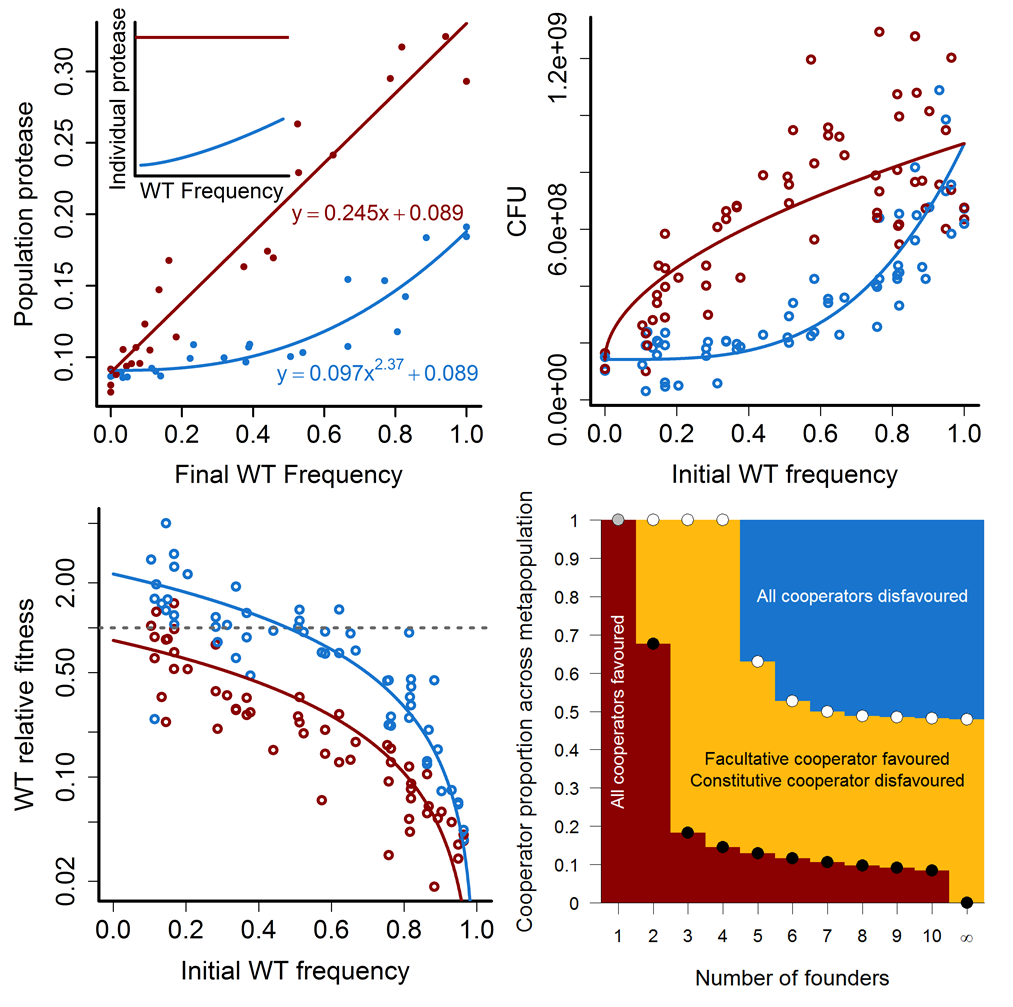
Figure S1: Protease production and competitive ability of wildtype cooperators when cooperation is experimentally fixed by adding QS signals in excess.** Signals (3-oxo-C12 and C4) were both added at a concentration of 45μM to generate data in red. Native response and competitive ability, without signal addition, are included in blue for comparison. Solid lines are fitted non-linear models. Topleft) Total protease production, as measured by an elastin congo red assay. Inset shows protease per WT individual. Topright) Population yield, measured as CFU/ 100μl. Bottomleft) WT relative fitness, plotted on log scale. Bottomright) Selection for cooperation in the metapopulation framework with varying numbers of founders, calculated using the solid curves for yield and relative fitness. QS regulated (facultative) cooperation is favoured over a much larger parameter space, including a well-mixed population (infinite number of founders). Dots represent equilibria for constitutive (black), QS-regulated (white), and either form of cooperation (grey).

**
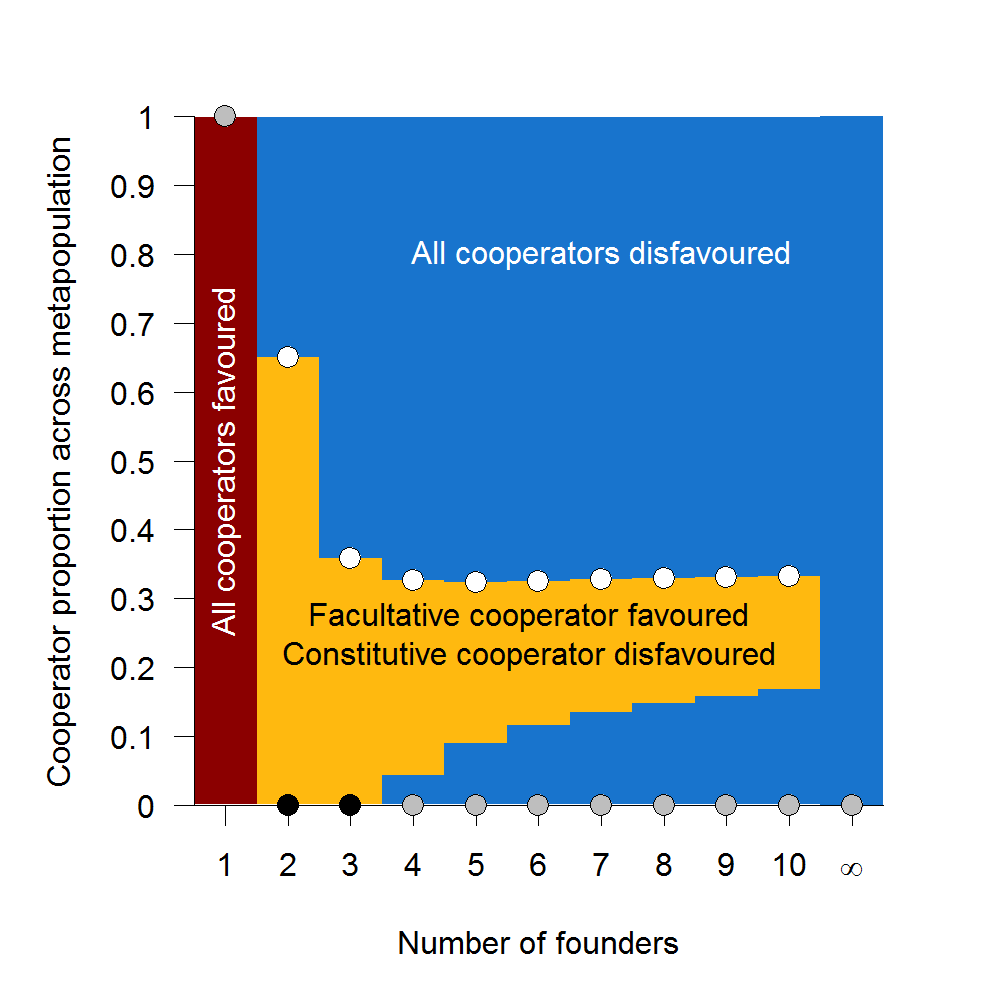
**

**Figure S2: Selection for cooperation in a metapopulation using our experimental data to allow cheats to cooperate at the optimal level (maximising their individual benefit).**Selection determined using statistical model fits shown in figure 3 and the metapopulation framework described in the methods. The cheat is considered to cooperate the amount that maximises their direct fitness (the maximum of the curve in Fig. 3c). Dots represent equilibria for constitutive (black), QS-regulated (white), and either form of cooperation (grey). Constitutive cooperation is only favoured when the population is completely structured (1 founder per subpopulation). QS regulated (facultative) cooperation is favoured over a much larger parameter space. For founder numbers of four and above bistability occurs in the system. An equilibrium of coexistence between cheats and facultative cooperators can be reached once facultative cooperators are above a threshold frequency, while below this frequency an equilibrium of only cheaters is favoured

**
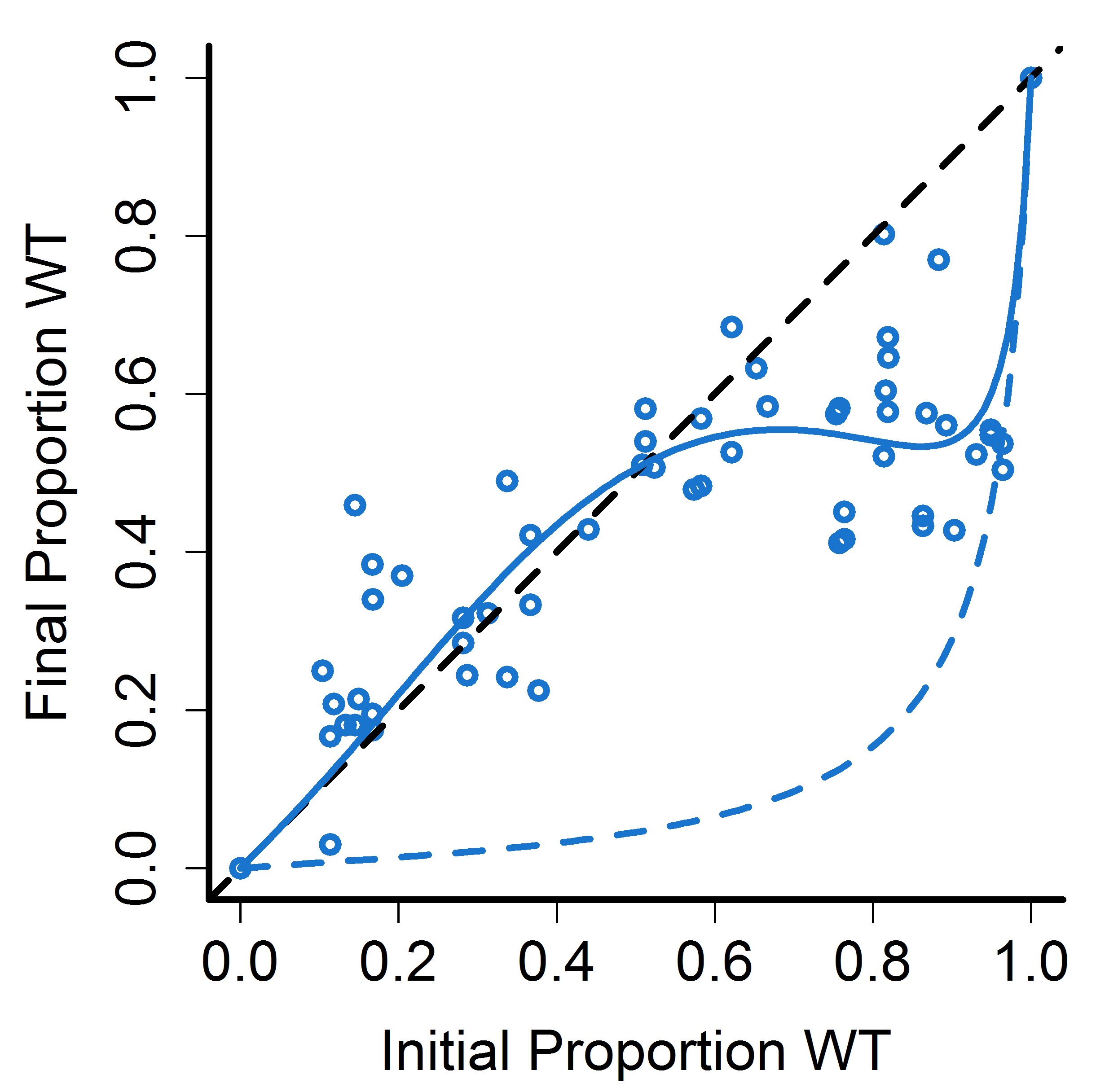
**

**Figure S3: Change in frequency of the WT strain over the course of 40 hours competition.** WT proportion was measured by plate counts at the start and end of competition, data in blue show competition under native control of cooperation. Sold curves show fits for the model fitted to the data in figure 3a, dashed lines show the predictions of the same model for constitutive cooperation.

**
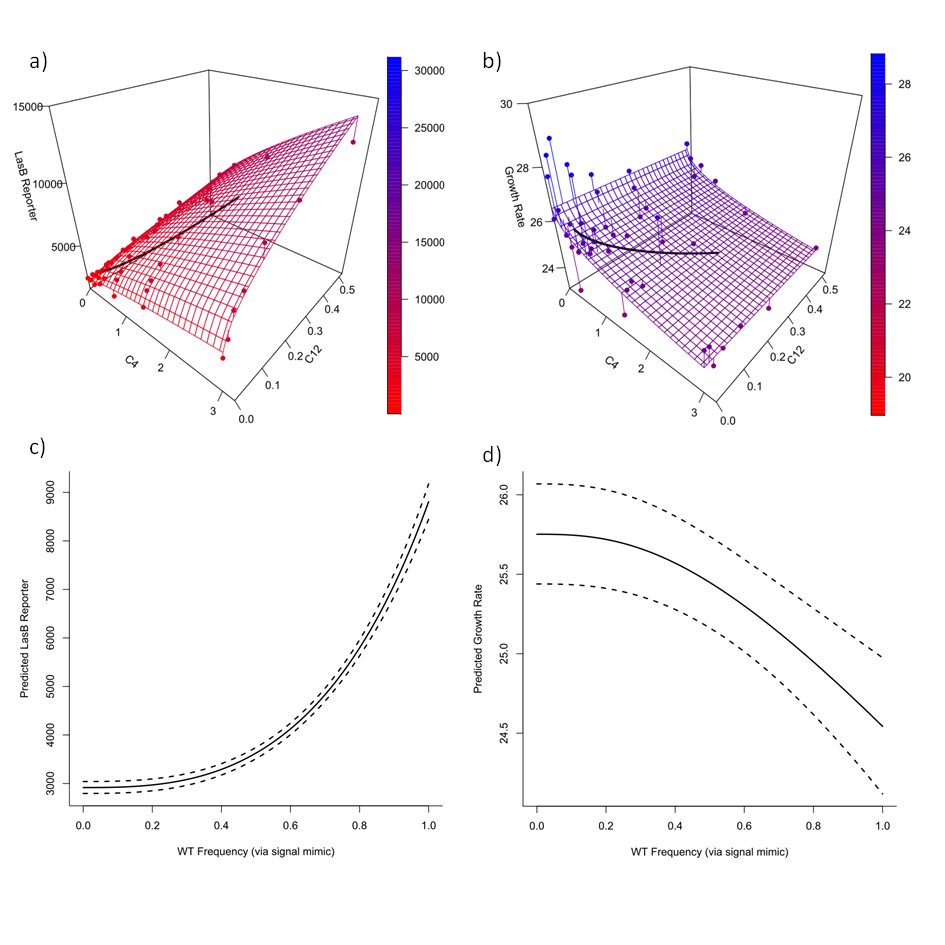
**

**Figure S4: The effect of QS activation on growth in an environment where protease is not group beneficial.** Pure cultures of double synthase (*ΔLasI ΔRhlI*) mutant were grown for 4 hours in QSM lacking the protein component. Quorum sensing was induced by the addition of C4 and 3-oxo-C12 signals to cultures at the start of growth. a) Luminescence measured from a LasB reporter after 4 hours growth, showing elastase expression increases in a diminishing manner with both C4 and 3-oxo-C12 signals. b) Growth rate of the synthase mutant over 4 hours growth, showing a diminishing cost with increasing concentration of C4 AHL. Black lines on surfaces show the path of signal concentrations measured and shown in figures 2a and 2b. The trajectory of these lines is shown in more detail in figure c and d. dashed lines are the 95% confidence intervals of the surface predictions.

**Protocol S1**

**Quorum sensing media recipe**

M9 Minimal media supplemented with: 1M CaCl_2_, 1M MgSO_4_, Hunters trace metals (Hutner *et al.*, 1950), 1% (w/v) bovine serum albumin and 0.1% (w/v) casamino acids. Filter sterilise (0.22μm filter).

**Experimentally enforcing constitutive cooperation**

For both the experiments measuring protease phenotype and competitive ability a second treatment was included which was treated identically except that 3-oxo-C12 HSL and C4 HSL were added at 45μM each (excess). Models were fitted to protease activity and yield using power law equations similar to the main text but models allowed all parameters to vary between the 2 treatments. For fitness data, power law models were fitted to cheat frequency (1-WT frequency), but otherwise model specification was the same. All models were then reduced to minimal models as in the main text. Relative fitness of the WT (v) was calculated as: X_1_(1-X_0_)/(X_0_ (1- X_1_)) where X_0_ and X_1_ are the proportions of WT cells at the start and end of competition respectively (Ross-Gillespie *et al.*, 2007).

With signal addition the exponent of the protease production curve was reduced compared to the native signal treatment (*H_0_* : Exponents the same between treatments, fitted difference = -1.31, s.e = 0.544, t_43_ = -2.398 , p < 0.05), and was no different from 1 (*H_0_* : Signal treatment exponent=1, fitted exponent = 0.95, s.e = 0.093, t_43_ = 0.580, p > 0.5). However although the amount of secreted enzyme produced by each individual WT was constant, secreted enzyme was produced at an increased rate compared with the native treatment (H_0_ : Effective slope the same between treatments, fitted difference = 0.15, s.e = 0.014, t_44_ = 10.582, p < 0.001). For yield (as measured by CFU), the exponent of the curve for the signal addition treatment was reduced (H_0_ : Exponents the same between treatments, fitted difference = -2.93, s.e = 0.568. t_111_ = -5.152, p < 0.001) but was significantly different from 1 (H_0_ : Exponent =1, fitted exponent = 0.53, s.e = 0.089, t_111_ = -5.30, p < 0.001). The maximal yield of the 2 treatments was not significantly different (H_0_ : Effective slope is the same between treatments, fitted difference = 1.45x10^8, s.e = 7.408x10^7, t_110_ = 1.961, p > 0.05 ).

For fitness data, the intercept (at a WT frequency of 1) was not significantly different between treatments (H_0_ : intercept is the same between treatments, fitted difference = -0.19, s.e = 0.151, t_107_ = -1.307, p > 0.1) or different from zero ( H_0_ : intercept = 0, fitted intercept = 0.01, s.e = 0.115, t_108_ = 0.115, p>0.5). However the effective slope (fitness at a WT frequency of 0) was significantly reduced with signal addition (H_0_ : Effective slope the same between treatments, fitted difference = -1.47, s.e = 0.199, t_109_ = -7.397, p< 0.001), and for both treatments the slope was significantly different from zero (H_0_ : Effective slope for native treatment = 0, fitted slope = 2.29, s.e = 0.184, t_109_ = 12.490 p < 0.01; H_0_ Effective slope for signal addition treatment = 0, fitted slope = 0.82, s.e = 0.136, t_109_ = 6.064, p < 0.001).

**Inducing variable levels of competition to measure costs of quorum sensing**

To measure costs of signalling a double synthase mutant (ΔLasI, ΔRhlI) was grown axenically in the QSM medium lacking the protein component, under otherwise identical conditions to the mixed populations. Both C4 and 3-oxo-C12 AHL signals were added to the cultures at the start of growth at a range of concentrations observed in figure 2a and 2b. After 4 hours the optical density (600nm) was measured and used to calculate growth rate (final density – initial density)/initial density. Elastase expression was measured using *LuxCDABE* cassette under the control of the *LasB* promoter (made using the mini CTXLux plasmid (Becher and Schweizer, 2000).

Maximal statistical models were fitted to log(growth rate) and squareroot (reporter luminescence) as multiple regressions on the squareroot of signal concentrations with an interaction term. Reported values are taken from minimal models. We see that protease expression (measured by a luminescent reporter, figure S4a) increases in a diminishing manner with concentration of both C4 (effect = 14.51, s.e = 1.36, t_45_ = 10.68, p < 0.001) and 3-oxo-C12 (effect = 23.00, s.e = 3.33, t_45_ = 6.91, p <0.001) AHL's. In addition there is a positive interaction between the two (effect = 29.27, s.e = 3.62, t_45_ = 8.08, p < 0.001). Quorum sensing activation has a cost in the growth rate (figure S4b) which is also diminishing with C4 concentration (effect = -0.056, s.e. = 0.010, t_47_ = -5.39,p < 0.001), although the effect of increasing C12 has no significant effect on growth. Using the signal environments at different proportions (taken from figure 2a and 2b) we can predict elastase expression (figure S4c) and growth rate (figure S4d) of the WT at different WT proportions in mixed populations. Dashed lines show the upper and lower confidence intervals on these predictions based on errors in the predictions in the surface from S4a and S4b.

**Dataset S1**: Data produced from phenotyping experiments, supplied as a separate file.

**Dataset S2:** Data produced from competition experiments, supplied as a separate file.

**Bibliography**

Becher A, Schweizer HP. (2000). Integration-proficient Pseudomonas aeruginosa vectors for isolation of single-copy chromosomal lacZ and lux gene fusions. *Biotechniques* **29**:948–+.

Hutner SH, Provasoli L, Schatz A, Haskins CP. (1950). Some Approaches to the Study of the Role of Metals in the Metabolism of Microorganisms. *Proc Am Philos Soc* **94**:152–170.

Ross-Gillespie A, Gardner A, West SA, Griffin AS. (2007). Frequency Dependence and Cooperation: Theory and a Test with Bacteria. *Am Nat* **170**:331–342.
